# Ferromagnetic Objects Magnetovision Detection System

**DOI:** 10.3390/ma6125593

**Published:** 2013-12-02

**Authors:** Michał Nowicki, Roman Szewczyk

**Affiliations:** Institute of Metrology and Biomedical Engineering, Warsaw University of Technology, Saint Andrzej Bobola street 8, Warszawa 02-525, Poland; E-Mail: r.szewczyk@mchtr.pw.edu.pl

**Keywords:** magnetovision, magnetic field imaging, security systems

## Abstract

This paper presents the application of a weak magnetic fields magnetovision scanning system for detection of dangerous ferromagnetic objects. A measurement system was developed and built to study the magnetic field vector distributions. The measurements of the Earth’s field distortions caused by various ferromagnetic objects were carried out. The ability for *passive* detection of hidden or buried dangerous objects and the determination of their location was demonstrated.

## 1. Introduction

Magnetovision utilizes the measurement of the distribution of magnetic field induction in a specific plane or in space and presenting it with a two dimensional (2D) image (for the plane) or 3D (for space). The word “magnetovision” comes from an analogy with thermal imaging, because the color in the magnetovision image corresponds to the magnetic flux density or the value of the magnetic field strength at a given point. It is also possible to obtain monochrome image in the form of isolines. The most suitable sensors for magnetovision are thin-film magnetoresistive sensors. They exhibit high sensitivity and have small size-typically 1 mm × 1 mm, or less [1]. Resolution of images depends directly on the number of measurement points per measurement line. The typical imaging device is a two-dimensional Cartesian coordinate system XY scanning device with Hall effect or magnetoresistive sensor, moving on the meandering path of a specific, usually rectangular area. In the case of an XY system with a single sensor, critical constraint affecting the measurement time is the number of lines along which the probe moves.

Magnetovision studies carried out previously were focused on the ability to measure stress in the ferromagnetic materials, in relation to the inverse magnetostrictive (Villari) effect [2]. By measuring the intensity of the magnetic field at the surface of samples subjected to mechanical stresses, good correlation of the magnetovision images and the stress distribution inside the test piece was obtained. This phenomenon open the new possibilities for non-destructive testing of the fatigue processes under cyclic mechanical stresses, also in the high frequency range. In case of the Villari effect, external magnetic field was not applied [3].

This paper presents an application of magnetovision method for passive detection and localization of dangerous metal objects. This method enables obtaining magnetovision images of unknown objects from a greater distance and on a larger surface area, which required the development of new methods for measuring and processing the results. The application of passive magnetovision system is vital, because the active metal detectors induction field can be sensed by specially constructed detonators. This applies particularly to the new generations of landmines and IED’s (improvised explosive device), reacting to the presence of active detectors, which presents a direct threat to minesweeper’s life [4].

## 2. Results and Discussion

**Figure 1:**
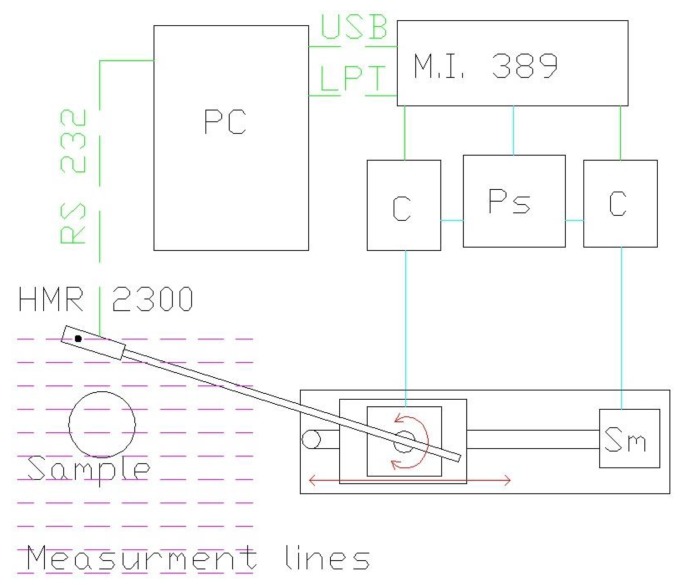
Schematic block diagram of the measuring system: HMR 2300—magnetoresistive sensor, Sample—investigated object, Sm—stepper motors, C—motor controllers, MI 3.8.9—trajectory generator, Ps—power supply, PC—controlling and image processing computer.

Ferromagnetic materials can be magnetized by the Earth’s magnetic field. Because their magnetic permeability is much higher than that of air, their presence creates a path of low reluctance, distorting lines of geomagnetic flux. Furthermore, most ferromagnetic elements have their own residual magnetism, which in effect causes them to act like a magnetic dipole. Thus, there are two sources of magnetization of a given ferromagnetic object—remanent magnetization and magnetization by external magnetic field such as earth magnetic field. Most ferromagnetic objects will disturb the surrounding magnetic field with both of these effects. Actual distinction between the sources of object magnetization, which in turn cause surrounding field perturbations, is unimportant from the detection and localization point of view. For the study of these disturbances, an XY scanning system was designed and built, with a single, tri-axial magnetoresistive Honeywell HMR2300 sensor (Figure 1). The sensor has a resolution of 7 nT and measuring range of ±200 µT, which is sufficient to measure weak magnetic fields.

During the measurements, no additional fields have been applied. Therefore only background disturbances were measured, mainly disturbances of the natural Earth’s magnetic field. Scanning probe system transit along parallel lines with a given interval (20 mm), setting the measuring plane (200 mm × 200 mm). Unlike other existing magneto-vision systems, application of tri-axial sensor enabled gathering information about the magnetic induction vector value and its direction with respect to each measurement point in the scanning plane, not only its absolute value, or value of only one component, as is the case with most Hall effect systems.

**Figure 2:**
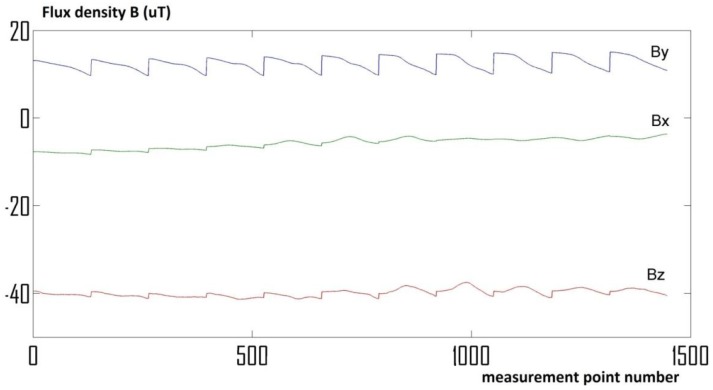
Raw measurements data collected using the magnetovision scanning system.

**Figure 3:**
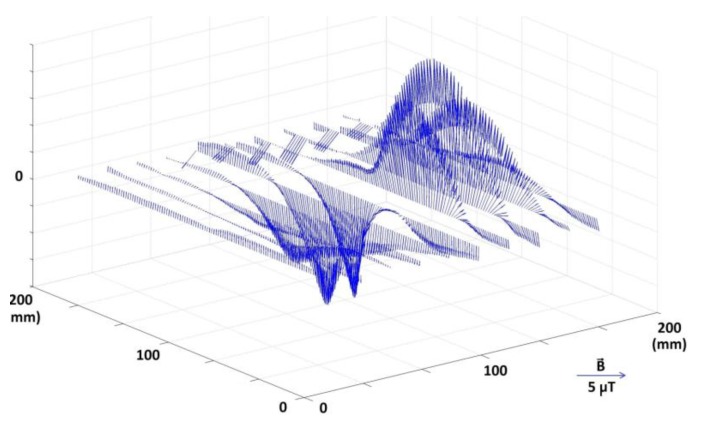
Visualization of induction vectors value and direction on measurement lines for simple dipole sample, with magnetic background removed. Additional disturbances of the magnetic field can be clearly seen.

**Figure 4:**
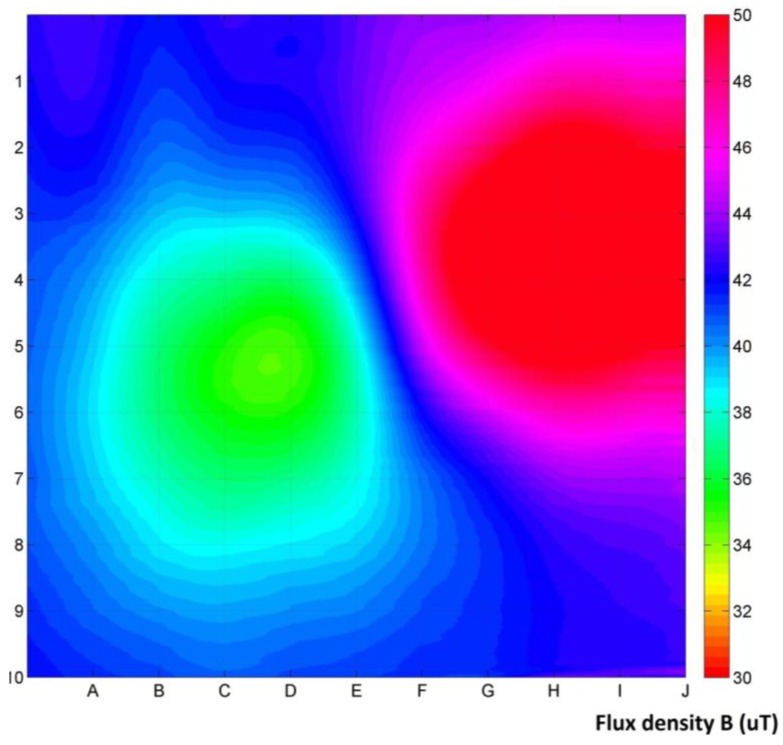
Visualization of the distribution of the magnetic induction vector absolute values, with the magnetic background intact. In addition to positive and negative disturbances, the Earth’s magnetic field can be seen.

Measurement results are gathered in the form of three signals, proportional to the induction vector components in measurement points (Figure 2). Raw data are enough to calculate the value and direction of the magnetic induction vectors in all measurement points. By subtraction of the relatively constant Earth’s magnetic field, vectors of additional magnetic disturbances can be visualized (Figure 3). Visualization of the distribution of the magnetic induction vector absolute values, interpolated to 1000 × 1000 points resolution, is also possible (Figure 4). 

Since the magnetoresistive sensor measures only the value of the three components of the flux density vector at a point in which it is physically located, a separation of distortion generated by a sample object from the background is problematic. The simplest laboratory solution is the differential measurement by measurement without the test object and subtracting the result from the measurement with an object. This method gives the best results, allowing precise separation of magnetic induction distribution of the background and the object, which allows for low-level noise in the magnetovision image. However, this method is possible only in certain conditions, where it is possible to make measurements with an object and without, in the same plane. For this reason, a method of differential measurement was developed, minimizing the impact of the background to the measurement results. With this method, both the Earth’s magnetic field as well as the other sources of background can be filtered out. In its basic form, a differential measurement is the measurement in two parallel planes, at the height *x* above the test object, and *x* + *h*, where h is the distance between the planes of measurement, and then subtracting the results.

Distribution of flux density lines near a ferromagnetic object placed in the Earth’s magnetic field is similar to a magnetic dipole field distribution. In particular, the magnetic flux density can be described as a dipole magnetic field characterized by a magnetic dipole moment $\vec{m}$ . Induction of the magnetic field on the axis of the magnet, in a vacuum, at a distance *x* from its center is expressed by the formula:

(1) $$\vec{B}=\frac{\mu_{0}}{2\pi x^{3}}\vec{m}=C\frac{1}{x^{3}}$$

where: $\vec{m}$ —magnetic dipole moment (Am^2^); $\mu_{0}=4\pi \cdot 10^{-7}\frac{H}{m}$ magnetic permeability of vacuum; $C=\frac{\mu_{0}}{2\pi }\vec{m}$ —induction replacement constant (Tm^3^) 

Since the value of the flux density *B* is reduced in proportion to the cube of the distance from the source, if *a* ≈ *x*, distortion $\vec{B_{1}}$ caused by the object in the first measurement plane will be up to 8 times greater than the $\vec{B_{2}}$ in the second plane. If, however, other sources of magnetic field are at a significantly greater distance *y* >> *x* from the first measurement plane, their influence $\vec{B_{B}}$ on the value of magnetic induction in the planes *P*_1_ and *P*_2_ will be similar. Therefore:

(2) $$\vec{B_{P1}}=\vec{B_{1}}+\vec{B_{B1}}$$

(3) $$\vec{B_{P2}}=\vec{B_{2}}+\vec{B_{B2}}$$

where: $\vec{B_{P1}}$ —result of flux density measurement in plane *P*_1_; $\vec{B_{P2}}$ —result of flux density measurement in plane *P*_2_; $\vec{B_{B1}}$ , $\vec{B_{B2}}$ —Background magnetic induction values in *P*_1_, *P*_2_ planes. Assuming: $\vec{B_{B1}}≅\vec{B_{B2}}$ , $\vec{B_{1}}>\vec{B_{2}}$ . Then:

(4) $$\vec{B_{P1}}-\vec{B_{P2}}\approx \vec{B_{1}}$$

As a result it is therefore possible to get a rough magnetovision image of the sample located a short distance from the sensor by subtracting the results of a measurement in the plane *P*_2_ from the results in the plane *P*_1_. Differential bi-plane measurement gives the absolute value of the difference in magnetic induction value between the measurement planes.

A similar method to compensate for the impact of background on the measurement result is the gradient measurement used in astrophysics and geology (e.g., in gradiometers). In the generalization it is based on the measurement of the magnetic field or gravity values at different levels and the field gradient designation on that basis. Use of this method also yields good results, but the images obtained are distinctly different than those obtained by the differential method. They allow us to distinguish between positive and negative areas of magnetic disturbance relative to the Earth’s field, but the bi-plane measurement gives a better picture of the silhouette of the hidden object. 

## 3. Experimental Section 

Measurements were carried out on a test stand setup described in previous chapter. For the purpose of the study, a steel cylinder, 70 mm in diameter and 20 mm in height was used as a sample object. Such element can simulate landmine for tests [5]. Distance between measurement planes for the differential method was set to *h* = 50mm.

**Figure 5:**
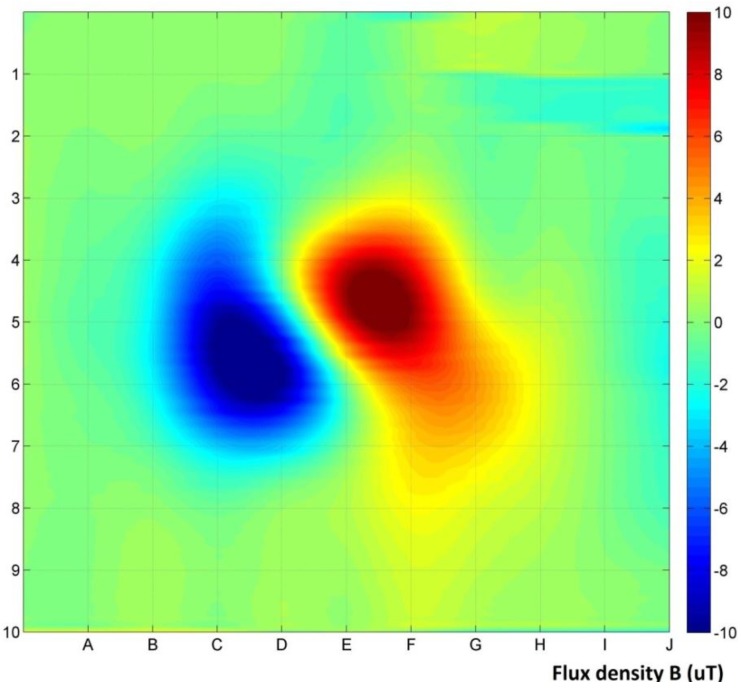
Gradient measurement results, 100 mm distance from the sample.

Figure 5 shows the gradient measurement results of the sample, with removing the influence of background magnetic field. Results exhibit a distinct difference between the positive and negative areas of magnetic disturbance. Distance of the sample from the plane of the measurement was *x* = 100 mm.

**Figure 6:**
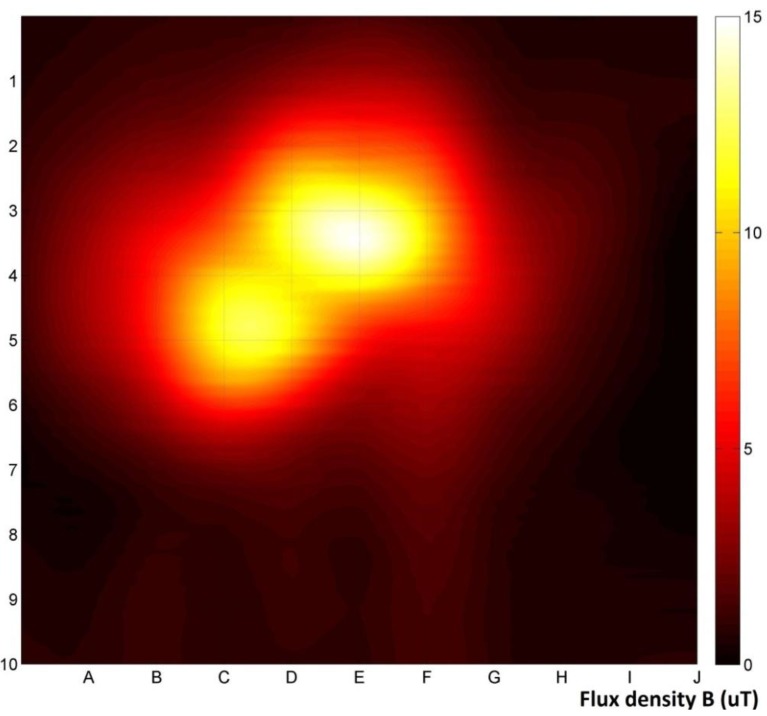
Differential bi-plane measurement within 100 mm of the sample.

Figure 6 shows a magnetovision image obtained by differential bi-plane measurement within 100 mm of the sample. Minimization of the background impact on the result is clearly visible. It is also clear, that this method provides the easiest way for localization of the sample, using the reference grid.

**Figure 7:**
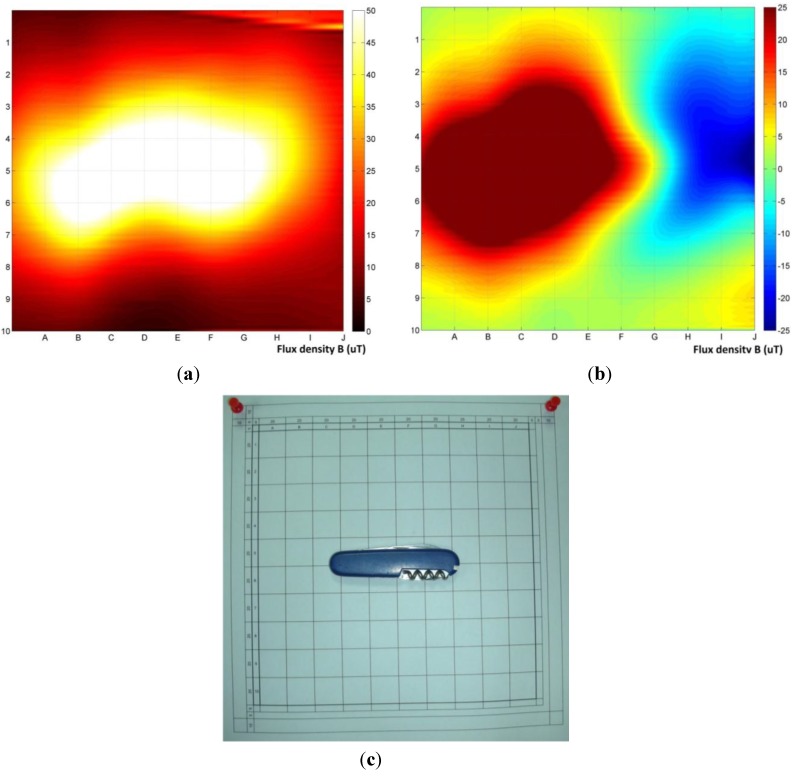
Measurement of the position of dangerous object (Swiss army knife): (**a**) biplane differential measurement; (**b**) gradient measurement without background separation; (**c**) a photograph of the actual position within 100 mm of the sample.

Figure 7 shows the results of the application of the developed method of measurements to determine the location of a sample, relative to the measurement plane. In addition, the comparison of the results of the differential bi-planar (Figure 7a) and gradient (Figure 7b) methods was performed. The object subjected to the test was a standard Swiss army knife, which is a very complex object from magnetic signature point of view. The distance of the first plane from the object was 50 mm. Bi-plane differential measurement results are shown in Figure 7a, whereas gradient measurement without removing the influence of the background is shown in Figure 7b. The position of sample on the reference grid is shown in Figure 7c. Results of up to 60 μT were obtained for absolute value of the disorders (Figure 7a) and −15–40 μT for the measurement of the background gradient (Figure 7b)

**Figure 8:**
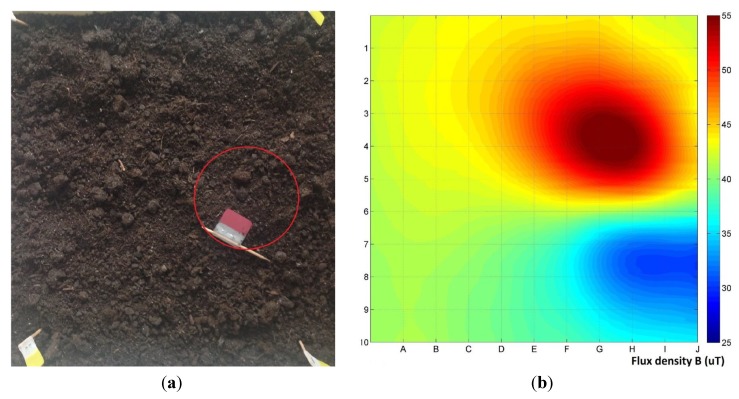
Application of the developed method of measurements to determine the location of a buried sample: (a) Comparison of the actual sample position (red circle); and (b) measurements results.

Figure 8 shows the results of the application of the developed method of measurements to determine the location of a sample, relative to the measurement plane. The object subjected to the test was buried under the 50 mm layer of soil. The distance of the measurement plane from the ground level was also 50 mm. For such a small distance, single plane measurement was enough to determine the position of sample. Part of the conducted experiments was to find the sample hidden from the operator, using only this method. Results of up to ±15 μT were obtained for absolute value of the disorders relative to the background gradient. Based on the results, the location and size of the object can be determined, which is very useful from practical point of view. 

## 4. Conclusions

Experimental setup for planar measurements of vector distribution of weak magnetic fields was developed. Moreover, new methodology of measurement, leading to decreasing the impact of magnetic background on the visualization of the results was presented. The developed methods allow a visualization of the distribution of the magnetic induction vector absolute values, its gradient as well as the value and direction of the magnetic flux density vector in different measurement points. The results obtained indicate that it is possible to passively detect and determine the location of dangerous ferromagnetic objects. This opens the new way to use magnetovision in public security systems, in particular for the detection of dangerous objects by police and sapper robots [6]. Unfortunately, the measured signals diminish with the distance from the object accordingly to Equation (1). Additionally, there is always some level of noise from the background, about 0.5 µT in the presented setup. Thus, the distance of clear detection of the object is now limited, as for instance doubling the distance will diminish the signal eightfold. Currently detection threshold for the knife sample is about 250 mm, but it can be improved with better signal processing and noise reduction techniques.
